# Investigation of the Synergistic Toxicity of Binary Mixtures of Pesticides and Pharmaceuticals on *Aliivibrio fischeri* in Major River Basins in South Korea

**DOI:** 10.3390/ijerph16020208

**Published:** 2019-01-13

**Authors:** In-Hyuk Baek, Youngjun Kim, Seungyun Baik, Jongwoon Kim

**Affiliations:** 1 Environmental Safety Group, Korea Institute of Science and Technology (KIST) Europe, Campus E 7.1, 66123 Saarbruecken, Germany; ih.baek@kist-europe.de (I.-H.B.); youngjunkim@kist-europe.de (Y.K.); sbaik@kist-europe.de (S.B.); 2 Center for Bioinformatics, Saarland University, Campus E 2.1, 66123 Saarbruecken, Germany; 3 Division of Energy and Environment Technology, KIST School, University of Science and Technology, Hwarang-ro 14-gil, Seoul 02792, Korea; 4 Chemical Safety Research Center, Korea Research Institute of Chemical Technology (KRICT), 141 Gajeong-ro, Yuseong-gu, Daejeon 34114, Korea

**Keywords:** mixture toxicity, concentration addition, pesticide, pharmaceuticals, *Aliivibrio fischeri*

## Abstract

This work introduces the potential synergistic toxicity of binary mixtures of pesticides and pharmaceuticals, which have been detected in substantial amounts in major river basins in South Korea. Different dose-response curve functions were employed in each experimental toxicity dataset for *Aliivibrio fischeri*. We tested the toxicity of 30 binary mixtures at two effect concentrations: high effect concentration [EC_50_] and low effect concentration (EC_10_) ranges. Thus, the toxicological interactions were evaluated at 60 effected concentration data points in total and based on model deviation ratios (MDRs) between predicted and observed toxicity values (e.g., three types of combined effects: synergistic (MDR > 2), additive (0.5 ≤ MDR ≤ 2), and antagonistic (MDR < 0.5)). From the 60 data points, MDRs could not be applied to 17 points, since their toxicities could not be measured. The result showed 48%-additive (*n* = 20), 40%-antagonistic (*n* = 17), and 12%-synergistic (*n* = 6) toxicity effects from 43 binaries (excluding the 17 combinations without MDRs). In this study, EC_10_ ratio mixtures at a low overall effect range showed a general tendency to have more synergistic effects than the EC_50_ ratio mixtures at a high effect range. We also found an inversion phenomenon, which detected three binaries of the combination of synergism at low concentrations and additive antagonism at high concentrations.

## 1. Introduction

Conventional chemical risk assessments frequently focus on individual substances rather than mixtures even though previous studies have shown that different mixture toxicities can results from combined effects among chemicals, even at no observed effect concentrations [1,2]. The types of the mixture toxicity are generally explained as additivity, synergism (greater than additivity), and antagonism (less than additivity) [3,4]. Among these combined effects, additive and synergistic toxicity effects can be regarded as more significant than the antagonistic effect (from the aspect of the regulatory chemical risk assessment, which assumes the worst-case scenario as the default) [5]. In this context, the conventional regulatory risk assessment of chemical mixtures is mostly based on the concentration addition (CA) model as a default for estimating the mixture toxicity [6,7,8,9]. Although the worst-case scenario can be substantial synergistic toxicity, the CA model, which ignores the synergistic interaction, has been mainly employed since available predictive models for estimating the synergistic effect have been very limited for the purpose of regulatory risk assessment [5]. In addition, some studies have shown that the synergistic interaction could be a relatively rare occurrence, at least within pesticide mixtures and realistic mixtures having low concentrations in mammals (which are comprised of approximately 5% of the tested mixtures) [10,11,12,13]. In contrast, a recently published review showed that less than 25% of research and experiments on the toxicity of chemical mixtures investigated seven or more compounds [14,15]. Some clinical researchers in human toxicology also showed that the probability of synergistic toxicity could be increased in proportion to the number of components, e.g., an 8% toxicity effect was caused by pharmaceutical products with five to ten mixed components, and a 38% toxicity effect was provoked by pharmaceuticals with fourteen or more mixture components [5,16,17]. This issue related to synergism is still controversial and difficult to conclude since most of the studies concerning mixture toxicity have been conducted with a specific binary mixture or simple mixtures having less than ten components [5]. Many studies have found that pesticides and pharmaceuticals were detected in the aquatic environment and thus they might lead to mixture toxicity for aquatic nontarget organisms [14,18,19,20,21]. Thus, when considering environmental mixtures with complex matrices and different chemicals, any potential risk posed might have been underestimated by the CA model if the synergistic interaction occurred in such mixtures [14]. 

The objective of this study was to preliminarily investigate the potential synergistic interactions of pharmaceutical and pesticide residues that can be found in the aquatic environment. For this purpose, we tested and evaluated the toxicity of different binary mixtures of pesticides and pharmaceuticals, which had been substantially identified in major river basins in South Korea. To evaluate the toxicity of single and mixed chemicals, a bioassay with a luminescent bacterium, *Aliivibrio fischeri,* which is widely used in standard toxicity methods, was conducted [22,23]. Based on the original best-fit approach [24], different dose-response curve (DRC) functions were employed to test data sets, and best-fit functions of single and mixed chemicals were used in the mixture toxicity modeling.

## 2. Materials and Methods 

### 2.1. Selection of Target Pesticides and Pharmaceuticals

Through previously published studies [25,26,27,28,29,30,31] that investigated the environmental concentration level of 47 pharmaceuticals in four major river basins (Han River, Geum River, Bukhan River, and Yeongsan River) in South Korea, 29 pharmaceuticals could be identified [32]. In addition, based on a study by Lee et al. [33] monitoring 140 pesticide residues in six Korean river basins (Han River, Geum River, Bukhan River, Yeongsan River, Mankyeong River, and Seomjin River), eight pesticides (isoprothiolane > butachlor > prothiofos > chlorpyrifos > hexaconazole molinate > diazinon and alachlor) could be found. From those 29 pharmaceuticals and eight pesticides, six chemicals, including four pharmaceuticals (tetracycline, trimethoprim, sulfamethoxazole, and chlortetracycline) and two pesticides (hexaconazole, and isoprothionlane), were finally selected as target mixutre components in this study (Table S1). The selction was made by considering their toxicities to *A. fischeri,* solubilities in water and carrier solvents, and detection frequencies in the aquatic environment. To our knowledge, this is the first study to investigate the toxicity of hexaconazole, isoprothiolane, and chlortetracycline and their binary mixtures to *A. fischeri*.

### 2.2. Test Reagents, Chemicals and Sample Preparation

Six target compounds were purchased from Sigma-Aldrich (Seelze, Germany). According to the physico-chemical properties of these compounds, stock solutions were prepared in either 99.9% dimethyl sulfoxide (DMSO, Sigma-Aldrich, Seelze, Germany) for trimethoprim and chlortetracycline or 99.8% ethanol (EtOH, Carl Roth GmbH, Karlsruhe, Germany) for tetracycline, hexaconazole, isoprothiolane and sulfamethoxazole. All of the stock solutions were kept at −20 °C under dark conditions until the working solutions were prepared. For quality control and quality assurance, the concentrations of the stock solutions were quantified with an HPLC system (Agilent 1290, Agilent Technologies, Santa Clara, CA, USA) connected to an Agilent triple quadrupole mass spectrometry (MS/MS) model 6460. Before the experiments, working solutions (1:25) were prepared by diluting the stock solutions in 2% sodium chloride for a marine bacterium, *A. vibrio*, according to ISO 11348-3 [22]. The pH values of the working solutions were checked and adjusted to between 6.0 and 8.0 with 1 N NaOH and 1 N HCl.

### 2.3. Testing Organism and Culture 

The bioluminescent bacteria *A. fischeri* (strain NRRL-B-11177 and formerly called *Vibrio fischeri*) were purchased from MicroTox^®^ (Lot number 15C4025A, Modern Water, New Castle, UK). The freeze-dried bacteria were activated with the reconstitution solution provided by the MicroTox^®^ for 30 mins at 15 °C. The activated bacteria were transferred to a photobacteria medium (Sigma-Aldrich) for preculture at 20 °C. For stock culturing, activated *A. fischeri* were estimated in a 250 ml main culture medium at an initial turbidity of a 1:10 dilution by UV-vis photometric (Ultraspec 3300, Amersham, Buckinghamshire, UK) at OD_578_ was 0.02 (10 formazine turbidity units, FNU). *A. fischeri* were cultured at 20 °C with shaking at 180 rpm/min until the turbidity of the OD_578_ was 1.74 (700 FNU to 1800 FNU). The amplified *A. fischeri* were purified twice with a 2% sodium chloride solution at 4 °C and 20 min at 7000 × g. The bacteria were slowly suspended in protective medium (66 g D-(+)-glucose monohydrate, 4 g sodium chloride, 2 g L-histidine and 0.5 g BSA in 100 mL) at an ice cooled condition until the turbidity of the OD_578_ was 2.58 (2000 FNU to 3000 FNU). The suspended stock bacteria were stored at −80 °C.

### 2.4. Single Chemical Toxicity Test 

Determination of an effective concentration of samples was performed [34] using the standardized methods of ISO 11348-3, 1998 by luminescent bacteria (*A. fischeri*) [22]. To activate, the frozen bacteria were suspended using a reconstituted solution (20 g sodium chloride, 0.3 g potassium chloride, HEPES 50 mM and glucose 50 mM for 1 liter) for 30 minutes at 15 °C. The activated luminescence bacteria were mixed with 2% sodium chloride at a 1:25 dilution. Six single compounds were serially diluted at a ratio of 1:1 (100 µL) on a flat-bottomed black 96-well plate (Greiner Bio-one, Kremsmünster, Germany). The bacterial suspensions were exposed to the serially diluted sample at a ratio of 100 µL by 100 µL. Then, reactive samples were measured after 15 min of exposure at 15 °C by a luminescent reader (Tristar2, Berthold technologies, Bad Wildbad, Germany). To assure the quality of the bacteria, a 100 ppm zinc sulfate solution (Sigma-Aldrich) was measured every time. 

### 2.5. Mixture toxicity test

An investigation of the synergistic toxicity of all binary mixtures that could be prepared from the six target compounds in Table S1 was conducted in a fixed ratio design based on the equitoxic mixture and the generation of dose-response curves from the mixtures employed in previous studies [35,36,37,38,39]. The advantages of the fixed ratio design are not only the ability to maximize the distribution of the effective dose range but also to minimize the number of experiments [37,40,41,42]. With the same compounds in Table S1, two different equitoxic mixtures were prepared at a 50% effective concentration for each compound as a high effective concentration ratio mixture (EC_50_ ratio mixture) to *A. fischeri* and at a 10% effective concentration ratio mixture (EC_10_ ratio mixture) as a low effective concentration ratio mixture. As shown in Table 1, a total of 30 equitoxic binary mixtures of each combination were tested at high and low effective concentration levels. However, the total doses of the mixtures were systematically different.

### 2.6. Statistical Analysis of the Mixture Toxicity 

A “best-fit” approach [24,39] was used to select the best model with the smallest sum of absolute residuals among the different sigmoidal functions because no single function could statistically describe all of the DRCs. Best-fit models with three-parameter sigmoidal equations were finally determined and applied to describe the experimental data of the mixture components tested in this study. The parameters in the sigmoidal regression equations and 95% confidence intervals were estimated using SigmaPlot^®^ (Ver. 12.5, Systat Software, Chicago, IL, USA). The ECx (e.g., EC_10_ and EC_50_) of the test chemicals was derived from the regression models shown in Table 2 and Table S2. It was assumed that all of the models were confined to the effects range from 0 to 100%. In order to compare between the highest and lowest toxicity without, we expressed the orders of magnitude which were determined as follows:

(1) $$\mathrm{Orders}\;\mathrm{of}\;\mathrm{magnitude}=\log(\frac{EC_{x-highest\;toxicity}}{EC_{x-lowest\;toxicity}})$$

However, in case a test chemical showed low solubility in water, the model for that chemical was assumed to have a range between >0% and <the maximum effect (%), where the chemicals were present at a maximum solubility in water under the test conditions in this study. The ECx for mixtures was calculated by the CA model according to the Loewe equation [11,43]:

(2) $$ECx_{\mathrm{mix}}={\left(\sum_{\overset{n}{i=1}}\frac{p_{i}}{ECx_{i}}\right)}^{-1}$$

where ECx_mix_ is the predicted effective concentration of a mixture; Pi and ECx_i_ are the fraction and the individual effective concentration of the component with in the mixture, respectively. 

MDR values (Belden et al., 2007 [11]) were used to quantify the interaction between the mixture components. The MDR values were frequently applied to determine the type of interactions of the mixture toxicity [11,13,44]. MDR is defined as:

(3) $$MDR=\frac{Predicted\;ECx\;of\;mixture}{Observed\;ECx\;of\;mixture}$$

where the predicted ECx indicates the effective concentration of a mixture based on the predictive model, and the observed ECx is the effective concentration of the mixture obtained from experimental toxicity testing. In this study, the CA model, which is recommended as a default approximation for mixtures, was used to predict mixture toxicity [5,10,11,45,46]. Based on the MDR value, the types of combined effects are divided into three groups: synergistic (MDR > 2), additive (0.5 ≤ MDR ≤ 2) and antagonistic (MDR < 0.5) [11,13]. 

## 3. Results and Discussion

### 3.1. DRCs for Single Compounds

**Figure 1:**
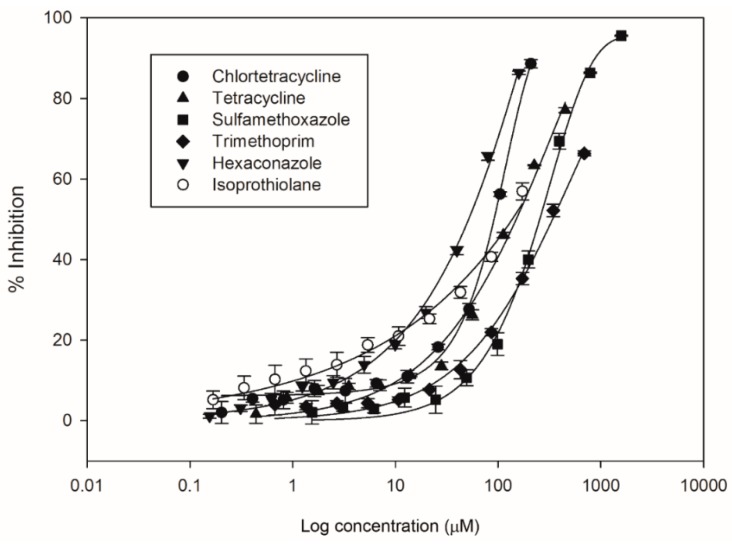
DRCs for the bioluminescent inhibition of *A. fischeri* for single compounds in Table 1 (the data points are geometric means ± standard deviation [SD] of the experimentally observed data and statistical best-fits (solid lines)).

DRCs of all six compounds in Table S1 were empirically determined for *A. fischeri,* as illustrated in Figure 1. 

Table 2 summarizes the parameter values of all the best-fitting regression models for the DRCs of single compounds. Table S2 explains the regression models described in Table 2. The best-fitting curves for all of the compounds showed high regression coefficients (r^2^) ranging from 0.969 to 0.995 and their ANOVA *p*-values were less than 0.0001. As presented in Table 2, the deviation between the steeper function of the highest slope values (65.25 for chlortetracycline) and the more gradual slope of the lowest (0.0034 for sulfamethoxazole) slope values was approximately 4.28 orders of magnitude. These considerable differences among the slopes of the DRCs of the compounds suggest that the compounds had highly different curve shapes for estimating the toxicity of each compound. The best-fitted DRCs for each compound had high regression coefficients (*r^2^*) ranging from 0.971 to 0.995. The EC50 values of *A. fischeri* were up to 0.82 orders of magnitude and ranged from 51.65 μM for hexaconazole to 338.81 μM for trimethoprim. However, the EC_10_ values of *A. fischeri* were up to 1.66 orders of magnitude and ranged from 1.05 μM for isoprothiolane to 47.79 μM for sulfamethoxazole. That is, hexaconazole and isoprothiolane presented the lowest effective concentrations (or the highest toxicity) at the respective EC_50_ and EC_10_ values, whereas isoprothiolane and sulfamethoxazole showed the highest effective concentrations (or the lowest toxicity) at the corresponding EC_50_ and EC_10_ values. These results also show that the toxicological profiles of compounds can be varied according to a given effective concentration level. In the cases of isoprothione, tetracycline, haxaconazole and trimethoprim, more than 80%-effect concentrations couldn’t be obtained under the testing conditions because of their water solubility limits. 

### 3.2. DRCs of Binary Mixtures 

**Figure 2:**
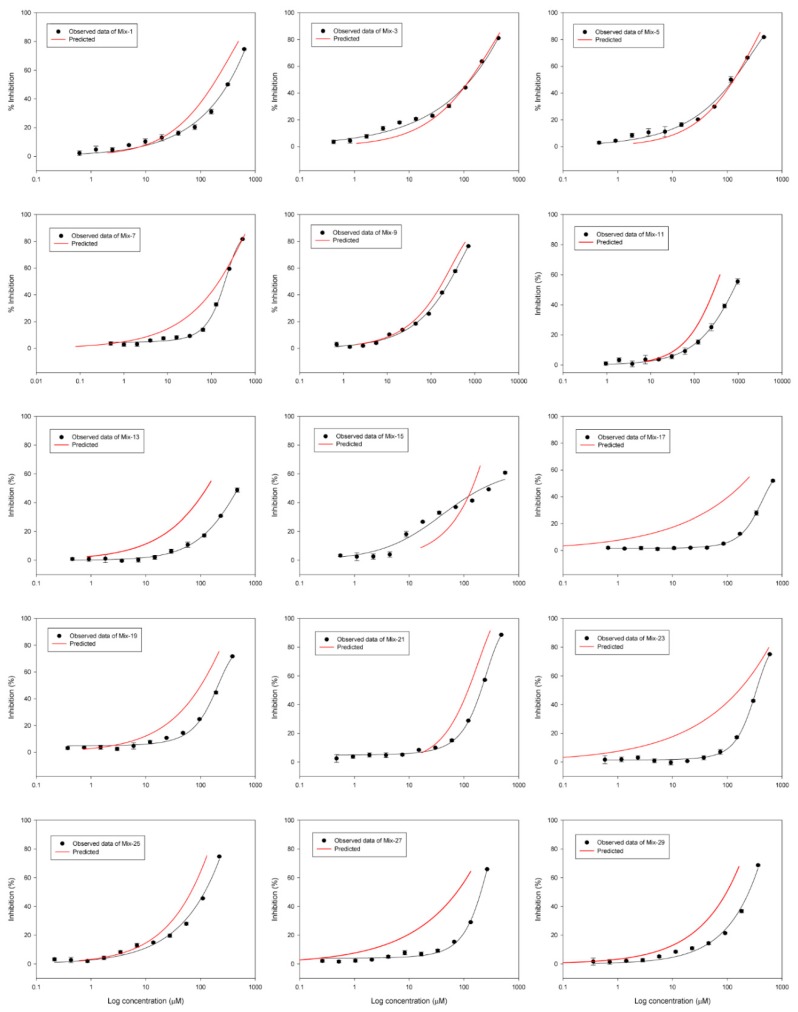
The DRCs for the observed bioluminescent inhibitions and the predicted inhibition (red lines) by the CA model for the binary equitoxic mixtures based on ratios at 50% effective concentrations for each component (the data points are geometric means ± standard deviation (SD) of experimentally observed data, and statistical best-fits for regression models are summarized in Table 3).

**Figure 3:**
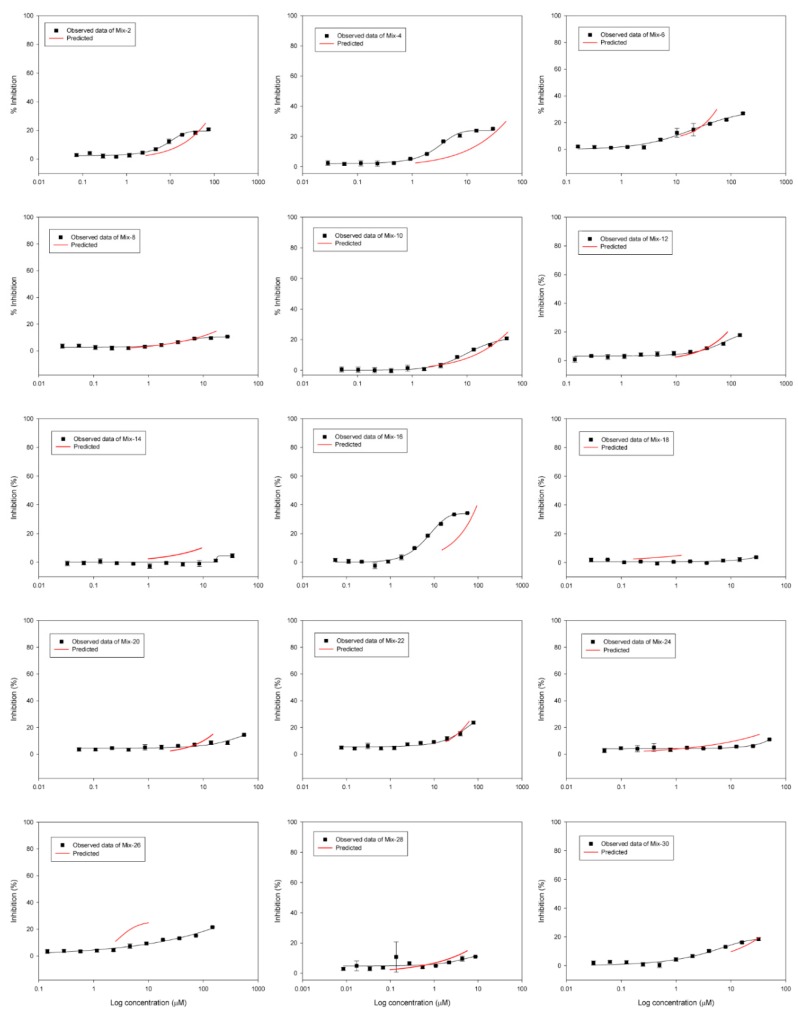
The DRCs for the observed bioluminescent inhibitions and the predicted inhibition (red lines) for the CA model for in the binary equitoxic mixtures and based on ratios at 10% effective concentrations for each component (the data points are geometric means ± standard deviation (SD)of experimentally observed data, and statistical best-fits for regression models are summarized in Table 3). The dose-response curves of 30 binary mixtures of pesticides and pharmaceuticals in Table 1, respectively.

The DRCs of binary mixtures in Table 1 were experimentally evaluated with high (EC_50_ + EC_50_) and low (EC_10_ + EC_10_) exposure levels (Figure 2; Figure 3). As shown in Table 3, best-fitting curves for all mixture combinations had high regression coefficients (r^2^) ranging from 0.817 to 0.997 except for four mixture combinations. The four exceptions are Mixture 14 (trimethoprim with hexaconazole; Mixture 14, r^2^ = 0.455), 18 (trimethoprim with isoprothiolane; Mixture 18, r^2^ = 0.429) 24 (*sulfa-methoxazole* with isoprothiolane; Mixture 24, r^2^ = 0.698) and 28 (hexaconazole with isoprothiolane; Mixture 28, r^2^ = 0.257). 

The probable reason for the high deviation of those two mixtures is that they were the EC_10_ ratio mixture, i.e., an equitoxic mixture based on ratios at 10% effective concentrations for each component but significantly less toxic than the others so that the ECx values could not be appropriately determined. As tested binary mixtures, the effective concentration data ranged from a low of 2.17 µmol/L (EC_10_ + EC_10_, tetracycline and hexaconazole) to a high of 779.94 µmol/L (EC_50_ + EC_50_, trimethoprim and sulfamethoxazole).

### 3.3. Statistical Analysis of Mixture Toxicity to Investigate Synergism 

Figure 2 and Figure 3 illustrate DRCs for the observed bioluminescent inhibitions and the predicted inhibition of *A. fischeri* by the CA model for the binary equitoxic mixtures based on ratios at 50% and 10% effective concentrations and following the combinations in Table 1, respectively. 

To quantify the toxicity interactions between mixture components, we calculated MDR values as shown in Table 4. Based on the MDR value, we strictly divided the three types of combined effects into synergistic (MDR > 2), additive (0.5 ≤ MDR ≤ 2) and antagonistic (MDR < 0.5) [13]. As shown in Figure 2 and Table 4, ten binaries of the EC_50_ ratio mixtures (i.e., EC*_50mix_*) showed the same interactions at two effective concentration values of EC_10_ and EC_50_. That is, four binaries of the EC*_50mix_* (Mixture 1, 5, 9, and 25) showed the additive effects at the EC_50_ and EC_10_ ranges. The antagonistic interaction of six binaries of EC*_50mix_* (Mixture 13, 17, 19, 23, 27, and 29) was then detected at the EC_50_ and EC_10_ ranges. Interestingly, five binaries of the mixture combination detected different interactions from the EC_50_ and EC_10_ ranges. Two binaries of the EC*_50mix_* (Mixture 3 and 15) found an additive interaction at the EC_50_ ranges and it resulted in a synergistic interaction at the EC_10_ ranges. At similar trend in one binary combination (Mixture 11) was observed for the antagonistic interaction at the EC_50_ ranges and an additive interaction at the EC_10_ ranges. In contrast, the two binaries of the EC*_50mix_* (Mixture 7, and 21) resulted in opposite trends in additive interaction at the EC_50_ ranges and antagonistic interaction at the EC_10_ ranges. These observed inversion phenomena of interaction between synergism at low concentrations and additive or antagonism at high concentrations are difficult to explain. As shown in Table 4, similar phenomena have been reported in previous studies [47,48,49,50]. Wang et al. [47] tested the spiramycin and ampicillin antibiotics on *Microcystis aeruginosa* at different equitoxic ratios. The study found the equivalent ratio (1:1) of the binary mixture of spiramycin and ampicillin showed a synergistic interaction at low concentrations and an antagonistic interaction at high concentrations.

In a study Nica et al. [48], they tested five veterinary pharmaceuticals for the interaction of synergistic, additive and antagonistic effects on *A. fischeri* by the combination index of the CA and IA models. They found inversion phenomena of antagonism at high concentrations and synergism at low concentrations from six binary combinations and one pentanary mixture with individual predicted no-effect concentrations. The authors assumed that these phenomena seemed to be independent of the mode of action, which are likely complex and mostly unknown in nature. Rodea-Palomares et al. [49] also found interesting results, i.e., opposite interactions between different aquatic organisms of cyanobacteria (*Anabaena* CPB 4337) and *A. fischeri* for three pharmaceuticals. The authors reported a tandemly changing interaction from antagonistic and additive effects at low effective concentration levels to synergistic effects at high effective concentration levels in an *A. fischeri* test for binary and tertiary mixtures.

As shown in Table 5, *Anabaena* tests showed a converse pattern against *A. fischeri* toxicity results. Ismael et al. carefully assumed that pharmaceuticals were shared by a common binding motif such as the same target and receptor sites. Because of the structural similarity, these unexpected interactions were shown between different aquatic organisms. Gonzalez-Pleiter et al. [50] also tested cyanobacteria (*Anabaena* CPB 4337) for levofloxacin and tetracycline. They found synergism at low effective concentration levels and antagonism at high effective concentration levels. Based on these results, we assume that the unexpected interaction was caused by tetracycline. In some cases, the interactions of experimental toxicity screening were different from predictive toxicity results with inversion phenomena of the interaction [47,48,49,50]. This observation suggests that the binary components could make the synergistic effects at low concentrations. Wang et al. [51] reported that the reaction mechanism differs between short-term (acute) and long-term (chronic) exposures because of quorum sensing molecules which known as *ain* and *lux* in *A. fischeri* [51]. So that better understanding the accurate synergistic interaction, chronic toxicity tests are required. In this study, the exposure levels of the tested chemicals were less than their environmentally relevant concentrations. Thus, further studies are needed to determine how substances as synergists interact with biomolecules at low concentrations from different model organisms including *A. fischeri.*

As shown in Figure 3 and Table 4, the reason for not available (i.e., n.a.) data indicates that they did not reach the EC_50_ and EC_10_ ranges. Thus, all binaries of the EC_10_ ratio mixtures (i.e., EC*_10mix_*) were not calculated for interactions from the binary mixture at EC_50_ ranges. Two binaries of EC*_10mix_* (Mixture 14 and 18) did not reach the experimental data at the EC_10_ ranges. Four binaries of the EC*_10mix_* (Mixture 2, 4, 16, and 30) showed synergistic interactions at the EC_10_ ranges, whereas seven additive interactions of EC*_10mix_* (Mixture 6, 8, 10, 12, 20, 22, and 26) and two antagonistic interactions of EC*_10mix_* (Mixture 24, and 28) occurred at EC_10_ ranges.

**Figure 4:**
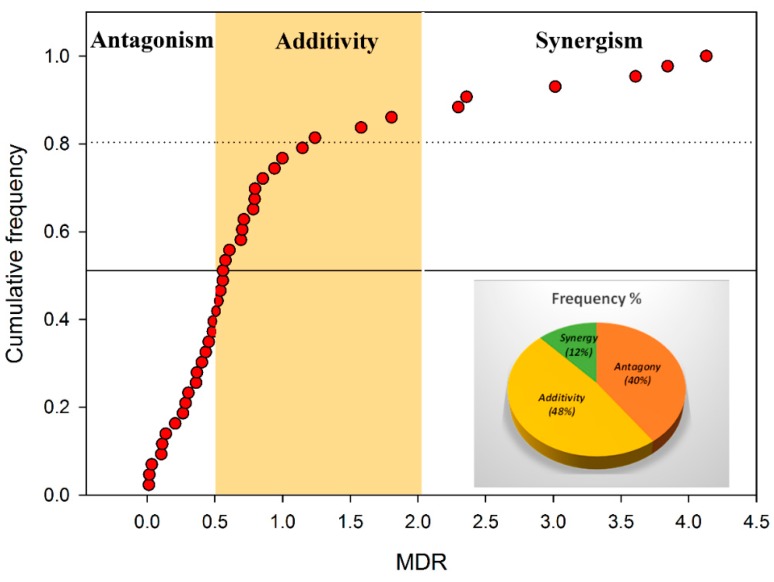
The cumulative distribution of model deviation ratios (MDRs) for quantifying the toxicity interactions of the binary mixtures of pharmaceuticals and pesticides (*n* = 43 from Table 4, excluding combinations without MDR) for *A. fischeri* (synergism: MDR > 2; additivity: 0.5 ≤ MDR ≤ 2; and antagonism: MDR < 0.5).

Figure 4 illustrates the cumulative distribution of MDRs. In total, the binary combinations of pharmaceuticals and pesticides detected in major river basins in Korea showed 48%-additive (*n* = 20 from Table 4 excluding combinations without MDR), 40%-antagonistic (*n* = 17), and 12%-synergistic (*n* = 6) toxicity effects from 43 binaries on the basis of the MDR values at high (EC_50_) and low effect (EC_10_) ranges. In this study, the EC_10_ ratio mixtures were at a low overall effect range and showed a general tendency to have more synergistic effects than EC_50_ ratio mixtures at the high effect range.

## 4. Conclusions

In this study, the toxicity of the six target compounds (e.g., four pharmaceuticals and two pesticides) detected in major river basins in South Korea and their binary mixtures (30 samples) were tested at high and low effect concentrations (e.g., EC50 and EC10 ratio mixtures) with luminescent bacterium *A*. *fischeri*. Thus, their toxicological interactions were evaluated at 60 effect concentration data points in total and based on model deviation ratios (MDRs) between predicted and observed toxicity values. The mixture toxicities of these mixtures were also predicted by the CA model to evaluate their toxicological interactions (e.g., additive, synergistic, and antagonistic effects) based on the MDR value. From the 60 data points, MDRs were not possible for 17 points since their toxicities could not be measured. The result showed 48%-additive (*n* = 20), 40%-antagonistic (*n* = 17), and 12%-synergistic (*n* = 6) toxicity effects from 43 binaries (excluding 17 combinations without MDRs). That is, from the mixture toxicity evaluation and modeling, we found twenty combinations of additive effects, seventeen combinations of antagonistic effects and six combinations of synergistic effects. In addition, we found inversion phenomena such as synergism at low concentrations and additive antagonism at high concentrations. The exposure levels of the tested chemicals were less than their environmentally relevant concentrations. Since the environmentally relevant concentrations of pesticides and pharmaceuticals detected in the aquatic environment can be present at low concentrations, further studies with different species need to be conducted to clarify the mechanisms, which can address what creates these inversion phenomena.

## Figures and Tables

**Table 1:** Binary mixture designs for target pesticides and pharmaceuticals.

Mixture No.	Substance A	Substance B	Mixture Design
1	Tetracycline	Sulfamethoxazole	EC_50_ + EC_50_
2	Tetracycline	Sulfamethoxazole	EC_10_ + EC_10_
3	Tetracycline	Hexaconazole	EC_50_ + EC_50_
4	Tetracycline	Hexaconazole	EC_10_ + EC_10_
5	Tetracycline	Chlortetracycline	EC_50_ + EC_50_
6	Tetracycline	Chlortetracycline	EC_10_ + EC_10_
7	Tetracycline	Isoprothiolane	EC_50_ + EC_50_
8	Tetracycline	Isoprothiolane	EC_10_ + EC_10_
9	Tetracycline	Trimethoprim	EC_50_ + EC_50_
10	Tetracycline	Trimethoprim	EC_10_ + EC_10_
11	Trimethoprim	Sulfamethoxazole	EC_50_ + EC_50_
12	Trimethoprim	Sulfamethoxazole	EC_10_ + EC_10_
13	Trimethoprim	Hexaconazole	EC_50_ + EC_50_
14	Trimethoprim	Hexaconazole	EC_10_ + EC_10_
15	Trimethoprim	Chlortetracycline	EC_50_ + EC_50_
16	Trimethoprim	Chlortetracycline	EC_10_ + EC_10_
17	Trimethoprim	Isoprothiolane	EC_50_ + EC_50_
18	Trimethoprim	Isoprothiolane	EC_10_ + EC_10_
19	Sulfamethoxazole	Hexaconazole	EC_50_ + EC_50_
20	Sulfamethoxazole	Hexaconazole	EC_10_ + EC_10_
21	Sulfamethoxazole	Chlortetracycline	EC_50_ + EC_50_
22	Sulfamethoxazole	Chlortetracycline	EC_10_ + EC_10_
23	Sulfamethoxazole	Isoprothiolane	EC_50_ + EC_50_
24	Sulfamethoxazole	Isoprothiolane	EC_10_ + EC_10_
25	Hexaconazole	Chlortetracycline	EC_50_ + EC_50_
26	Hexaconazole	Chlortetracycline	EC_10_ + EC_10_
27	Hexaconazole	Isoprothiolane	EC_50_ + EC_50_
28	Hexaconazole	Isoprothiolane	EC_10_ + EC_10_
29	Chlortetracycline	Isoprothiolane	EC_50_ + EC_50_
30	Chlortetracycline	Isoprothiolane	EC_10_ + EC_10_

**Table 2:** Parameters of the regression models for dose-response curves of *A. fischeri* for pesticide and pharmaceutical single compounds in Table S1 (the 95% confidence intervals are provided in the brackets).

Substance	EC_50_ (μM)	EC_10_ (μM)	RM ^1^	r^2^	Model Parameter		
					A ^2^	Β ^3^	Γ ^4^
Hexaconazole	51.65 (50.97–52.33)	3.06 (2.38–3.74)	C	0.995	1.4335	0.0035	0.5869
Isoprothiolane	137.07 (136.14–138.0)	1.05 (0.12–1.85)	H	0.971	97.3866	0.3312	1.10 × 10^9^
Tetracycline	150.08 (148.85–151.30)	10.60 (9.38–11.83)	L	0.981	1.4806	–0.7364	374.5933
Trimethoprim	338.81 (338.05–339.56)	26.20 (25.45–26.95)	L	0.990	1.2286	–0.7997	542.4834
Sulfamethoxazole	254.20 (253.25–255.15)	47.79 (46.83–48.74)	C	0.994	0.9561	0.0034	1.1932
Chlortetracycline	91.32 (90.52–92.12)	12.32 (11.52–13.12)	G	0.993	0.9902	65.2541	66.4673

Notes. ^1^ Regression models (C: Chapman, G: Gompertz, H: Hill, L: Logistic); ^2^ Height; ^3^ Slope; and ^4^ Center point.

**Table 3:** Parameters of regression models for dose-response curves of 30 binary mixtures of pesticides and pharmaceuticals in Table 1 (the 95% confidence intervals are provided in the brackets).

Mixture No.	EC_50_ (μM)	EC_10_ (μM)	RM ^1^	r^2^	Model Parameter		
					A ^2^	Β ^3^	Γ ^4^
1	313.95 (313.07–314.82)	16.62 (15.74–17.50)	C	0.987	2528.4458	2.47 × 10^−6^	0.5477
2	n.a.^5^	7.43 (6.99–7.88)	G	0.968	19.5735	6.4890	4.8497
3	133.58 (132.69–134.46)	2.89 (2.01–3.78)	C	0.989	915.2173	7.38 × 10^−6^	0.4200
4	n.a.	2.17 (1.75–2.59)	G	0.983	23.9224	2.0367	1.8951
5	129.70 (128.75–130.66)	5.98 (5.02–6.93)	C	0.989	102.0025	0.0025	0.5494
6	n.a.	9.54 (8.83–10.25)	L	0.954	30.2768	−0.8861	21.1859
7	203.46 (202.88–204.04)	36.83 (36.25–37.41)	G	0.996	84.6072	118.9205	127.0557
8	n.a.	11.66 (11.25–12.06)	G	0.874	10.4177	3.3048	1.0944
9	265.16 (264.62–265.69)	15.55 (15.01–16.09)	C	0.996	99.5885	0.0015	0.6049
10	n.a.	8.64 (8.18–9.10)	L	0.970	22.0641	−1.4308	9.8530
11	779.94 (779.31–780.56)	62.99 (62.36–63.61)	C	0.989	83.7765	0.0009	0.7231
12	n.a.	50.31 (49.80–50.81)	G	0.914	19.2041	49.3054	29.2598
13	488.97 (488.44–489.49)	59.282 (58.76–59.81)	H	0.991	131.1544	0.9527	812.9558
14	n.a.	n.a.	G	0.455	4.5523	0.5916	17.0836
15	222.30 (220.95–223.65)	4.56 (3.22–5.91)	H	0.964	63.8649	0.7633	41.4132
16	n.a.	3.91 (3.38–4.44)	C	0.988	33.8541	0.1489	1.4924
17	637.32 (637.01–637.64)	154.31 (153.99–154.63)	G	0.997	60.7316	216.8352	282.2291
18	n.a.	n.a.	G	0.429	8.9924	25.6941	25.7707
19	209.14 (208.45–209.83)	34.75 (34.06–35.44)	G	0.992	78.4915	114.7884	117.7289
20	n.a.	26.94 (26.43–27.44)	G	0.817	17.7472	30.9625	9.7285
21	206.10 (205.57–206.64)	37.41 (36.87–37.95)	G	0.997	96.2390	135.9703	148.5324
22	n.a.	15.85 (15.31–16.38)	G	0.9331	27.4285	34.3506	16.1526
23	339.80 (339.23–340.37)	106.78 (106.22–107.36)	G	0.9951	83.3055	163.6608	229.7587
24	n.a.	45.77 (45.31–46.24)	S	0.6981	1804.3798	51.2073	311.5280
25	119.13 (118.31–119.96)	8.40 (7.57–9.23)	H	0.9884	1.37 × 10^5^	0.6069	5.52 × 10^7^
26	n.a.	13.89 (13.46–14.33)	C	0.9560	158.6840	9.12 × 10^-6^	0.3080
27	204.18 (203.53–204.83)	45.75 (45.10–46.40)	G	0.9901	107.4683	139.9025	166.7380
28	n.a.	5.82 (4.61–7.04)	G	0.2571	12.1655	3.7398	–0.2706
29	247.79 (246.98–248.59)	28.44 (27.64–29.24)	H	0.9870	4.73 × 10^5^	0.7435	5.52 × 10^7^
30	n.a.	4.38 (3.84–4.91)	C	0.9435	18.6241	0.1128	0.6594

Notes. ^1^ Regression models (C: Chapman, G: Gompertz, H: Hill, L: Logistic); ^2^ Height; ^3^ Slope; and ^4^ Center point, ^5^ Not available.

**Table 4:** Observed and predicted EC*x_mix_* values of tested mixtures of pharmaceuticals and pesticides in binary combinations, and MDR values to address the interactions between components (the 95% confidence intervals are provided in the brackets).

Mixture No.	EC_50mix_^1^				EC_10mix_			
	Observed (EC_50_, μM)	Predicted ^2^	MDR ^3^	Type ^4^	Observed (EC_10_, μM)	Predicted	MDR	Type
EC_50_ ratio mixtures ^5^								
1	313.95 (313.07–314.82)	169.63	0.54	Add.^6^	16.62 (15.74–17.50)	13.55	0.82	Add.
3	133.58 (132.69–134.46)	128.47	0.96	Add.	2.89 (2.01–3.78)	8.71	3.01	Syn.
5	129.70 (128.75–130.66)	132.37	1.02	Add.	5.98 (5.02–6.93)	10.92	1.83	Add.
7	203.46 (202.88–204.04)	147.27	0.72	Add.	36.83 (36.25–37.41)	3.84	0.10	Anta. ^7^
9	265.16 (264.62–265.69)	189.17	0.71	Add.	15.55 (15.01–16.09)	13.61	0.88	Add.
11	779.94 (779.31–780.56)	281.92	0.36	Anta.	62.99 (62.36–63.61)	36.51	0.58	Add.
13	488.97 (488.44–489.49)	129.65	0.27	Anta.	59.282 (58.76–59.81)	8.20	0.14	Anta.
15	222.30 (220.95–223.65)	139.68	0.63	Add.	4.56 (3.22–5.91)	16.45	3.61	Syn. ^8^
17	637.32 (637.01–637.64)	194.16	0.30	Anta.	154.31 (153.99–154.63)	2.00	0.01	Anta.
19	209.14 (208.45–209.83)	101.47	0.49	Anta.	34.75 (34.06–35.44)	7.21	0.21	Anta.
21	206.10 (205.57–206.64)	119.87	0.58	Add.	37.41 (36.87–37.95)	17.01	0.45	Anta.
23	339.80 (339.23–340.37)	167.07	0.49	Anta.	106.78 (106.22–107.36)	1.70	0.02	Anta.
25	119.13 (118.31–119.96)	66.93	0.56	Add.	8.40 (7.57–9.23)	5.04	0.60	Add.
27	204.18 (203.53–204.83)	74.93	0.37	Anta.	45.75 (45.10–46.40)	1.57	0.03	Anta.
29	247.79 (246.98–248.59)	100.21	0.40	Anta.	28.44 (27.64–29.24)	3.18	0.11	Anta.
EC_10_ ratio mixtures								
2	n.a. ^9^	n.a.	-	-	7.43 (6.99–7.88)	17.53	2.36	Syn
4	n.a.	n.a.	-	-	2.17 (1.75–2.59)	8.96	4.13	Syn.
6	n.a.	n.a.	-	-	9.54 (8.83–10.25)	12.03	1.26	Add.
8	n.a.	n.a.	-	-	11.66 (11.25–12.06)	8.57	0.73	Add.
10	n.a.	n.a.	-	-	8.64 (8.18–9.10)	13.84	1.60	Add.
12	n.a.	n.a.	-	-	50.31 (49.80–50.81)	41.16	0.82	Add.
14	n.a.	n.a.	-	-	n.a.	n.a.	-	-
16	n.a.	n.a.	-	-	3.91 (3.38–4.44)	15.03	3.84	Syn.
18	n.a.	n.a.	-	-	n.a.	n.a.	-	-
20	n.a.	n.a.	-	-	26.94 (26.43–27.44)	14.00	0.52	Add.
22	n.a.	n.a.	-	-	15.85 (15.31–16.38)	18.52	1.17	Add.
24	n.a.	n.a.	-	-	45.77 (45.31–46.24)	12.98	0.28	Anta.
26	n.a.	n.a.	-	-	13.89 (13.46–14.33)	11.18	0.80	Add.
28	n.a.	n.a.	-	-	5.82 (4.61–7.04)	2.52	0.43	Anta.
30	n.a.	n.a.	-	-	4.38 (3.84–4.91)	10.07	2.30	Syn.

Note. ^1^ *ECx_mix_*: effective concentrations of a mixture causing *x%* toxicity effect; ^2^ Values predicted by the concentration addition model; ^3^ Model deviation ratio; ^4^ Type of combined toxic effects; ^5^ *ECx* ratio mixture: an equitoxic mixture based on ratios at *x*% effective concentrations for each component; ^6^ Additivity; ^7^ Antagonism; ^8^ Synergism; and ^9^ Not available.

**Table 5:** A summary of the studies related to the interaction of inversion phenomena.

Mixture	Experimental Design	Species	Endpoint	Convind Effect		Quantification Methods	Ref.
				High Level	Low Level		
Two antibiotics	Binary equitoxic mixture ratio (5:1, 1:1, 1:5)	Bacteria *Microcystis aeruginosa* (MA)	EC50 and EC5 for MA cell from equitoxic ratio SP/Amp (5:1, 1:5, 1:1)	Antagonism 1:1 ratio >0.7 ug/L	Synergism 1:1 ratio <0.5 ug/L	Departure from additivity model (CA, IA)	[47]
Five veterinary pharmaceuticals	Binary and multicomponent mixture	Bacteria *A. fischeri*	Applying the combination index method from active pharmaceutical compound interactions for bacteria	Antagonism	Synergism	Departure from combination index (CA, IA)	[48]
			Diclofenac : Sulfamethizole	EC_50_ 1.13	EC_10_ 0.61		
			Acetylsalycilic acid : Sulfamethizole	EC_50_ 2.58	EC_10_ 0.85		
			Chlortetracycline : Amoxicillin	EC_50_ 2.16	EC_10_ 0.08		
			Acetylsalycilic acid : Diclofenac	EC_50_ 1.13	EC_10_ 0.73		
			Sulfamethizole : Amoxicillin	EC_50_ 1.57	EC_10_ 0.41		
			Acetylsalycilic acid : Amoxicillin	EC_50_ 2.17	EC_10_ 0.72		
			Predicted no-effect concentration (five pharmaceutical compound mixture)	EC_50_ 1.36	EC_10_ 0.61		
Three pharmaceuticals	Binary and ternary combinations	Bacteria *A. fischeri*Cyanobacteria *Anabaena* CPB4337	Applying the combination index with isobologram equation methods from pharmaceutical compounds for in vitro and in vivo bioassay	Antagonism	Synergism	Departure from combination index (CA and IA) with isobologram equation	[49]
			Fenofibrate : Bezafibrate	EC_90_ 2.59 EC_50_ 1.19	EC_10_ 0.55		
			Fenofibrate : Gemfibrozil	EC_90_ 12.9 EC_50_ 1.29	EC_10_ 0.13		
			Fenofibrate : Gemfibrozil : Bezafibrate	EC_90_ 3.92	EC_50_ 0.57 EC_10_ 0.09		
Five antibiotics	Binary and multicomponent mixture	Cyanobacteria *Anabaena* CPB4337 Microalgae *Raphidocelis subcapitata*	Applying combination index with isobologram equation methods from pharmaceutical compound for in vitro and in vivo bioassay	Antagonism	Synergism	Departure from combination index (CA and IA) with isobologram equation	[50]
			Levofloxacin : Tetracycline	EC_50_ 1.6	EC_10_ 0.37		

## Supplementary Materials

The following are available online at http://www.mdpi.com/1660-4601/16/2/208/s1, Table S1: Selected pesticides and pharmaceuticals, which were identified in major river basins in South Korea, Table S2: The regression models employed in describing the dose-response curves in this study.
