# Supplementary material for: Investigation of the Synergistic Toxicity of Binary Mixtures of Pesticides and Pharmaceuticals on Aliivibrio fischeri in Major River Basins in South Korea
Source: Int J Environ Res Public Health. 2019 Jan 13;16(2):208. doi: 10.3390/ijerph16020208 (PMC6352224; doi:10.3390/ijerph16020208)
Supplement: Supplementary file 1 [file ijerph-16-00208-s001.pdf]

Supplementary Materials

Investigation of the Synergistic Toxicity of Binary Mixtures of Pesticides and Pharmaceuticals on *Aliivibrio fischeri* in Major River Basins in South Korea

In-Hyuk Baek [1,2], Youngjun Kim [1,3], Seungyun Baik [1] and Jongwoon Kim [1,3,4,*]

Table S1. Selected pesticides and pharmaceuticals, which were identified in major river basins in South Korea

Substance	CAS RN	Structure	MW	Type	Use	Reference
Chlortetracycline	57-62-5	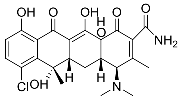	478.88	Pharmaceutical	Veterinary and human medicine	[34–39]
Hexaconazole	79983-71-4	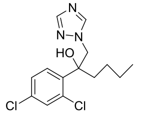	314.21	Pesticide	Fungicide	[40]
Isoprothiolane	50512-35-1	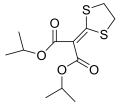	290.40	Pesticide	Fungicide	[40]
Sulfamethoxazole	72-14-0	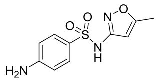	255.32	Pharmaceutical	Human medicine (Antibiotic)	[34,37–39,41]
Tetracycline	60-54-8	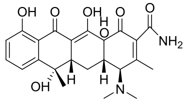	444.43	Pharmaceutical	Veterinary and human medicine	[34,35,39,42]
Trimethoprim	738-70-5	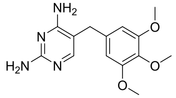	290.32	Pharmaceutical	Human medicine (Antibiotic)	[34,37–39,41]

Table S2. The regression models employed in describing the dose-response curves in this study

Regression model	Function
Gompertz (G)	$E(c) = \alpha\ (\exp\left(-\exp\left(-\left(\frac{-c-\gamma}{\beta}\right)\right)\right))$
Sigmoid (S)	$E(c) = \frac{\alpha}{1+\exp\left(-\frac{c-\gamma}{\beta}\right)}$
Logistic (L)	$E(c) = \frac{\alpha}{1+\left(\frac{c}{\gamma}\right)^{\beta}}$
Hill (H)	$E(c) = \frac{\alpha c^{\beta}}{\gamma^{\beta}+c^{\beta}}$
Chapman (C)	$E(c) = \alpha\left(1-exp(-\beta c)\right)^{\gamma}$

Notes. E(c): the fractional effect elicited at concentration c; α, β, and γ: parameters of regression models (corresponding statistical estimates).
